# Phenotypic characterization of ESBL-producing urinary isolates of *E. coli* and *Klebsiella* spp. in a tertiary care children's hospital in Nepal

**DOI:** 10.1186/s41182-024-00587-3

**Published:** 2024-03-01

**Authors:** Santosh Pantha, Hiramani Parajuli, Charu Arjyal, Shovana Thapa Karki, Dhiraj Shrestha

**Affiliations:** 1 Department of Microbiology, Tri-Chandra Multiple Campus, Kathmandu, Nepal; 2 Center for Climate and One Health Research (CCOHR), Kathmandu, Nepal; 3 Department of Pathology, International Friendship Children Hospital, Kathmandu, Nepal; 4 Department of Microbiology, Shi-Gan International College of Science and Technology (SICOST), Kathmandu, Nepal; 5 Department of Microbiology, Padma Kanya Multiple Campus, Kathmandu, Nepal

**Keywords:** Children, *E. coli*, ESBL, *Klebsiella*, Nepal, UTI

## Abstract

**Background:**

The production of extended-spectrum beta-lactamases (ESBLs) among uropathogens, particularly *E. coli* and *Klebsiella* spp., poses a severe public health concern. This study explored the epidemiology of ESBL-producing *E. coli* and *Klebsiella* spp. isolated from urine samples obtained at a tertiary care children's hospital in Nepal.

**Methods:**

A cross-sectional study was conducted from August 2016 to February 2017. A total of 745 clean catch urine samples were obtained from pediatric patients under the age of 13 and subjected to semiquantitative culture. *E. coli* and *Klebsiella* spp. were identified using standard laboratory protocols. Antibiotic susceptibility testing was performed using the Kirby-Bauer disc diffusion method, and ESBL producers were phenotypically identified using the combined disk method.

**Results:**

Among the bacterial isolates, *E. coli* predominated, accounting for 139 (81.8%) positive cases. Notably, *E. coli* showed high susceptibility to nitrofurantoin, with 117 (84.2%) isolates being susceptible. Meanwhile, *K. pneumoniae* showed high susceptibility to gentamicin, with 21 (91.3%) isolates being susceptible. Of the 163 isolates of *E. coli* and *Klebsiella* spp., 62 (38.0%) were identified as multidrug-resistant (MDR), with 42 (25.8%) confirmed as phenotypic ESBL producers. Remarkably, all 41 (100%) ESBL-producing *E. coli* isolates were susceptible to imipenem.

**Conclusions:**

The prevalence of ESBL producers among *E. coli* and *K. pneumoniae* isolates from pediatric patients underscores the importance of antimicrobial stewardship. Nitrofurantoin and gentamicin emerge as effective empirical treatment choices against these pathogens in children. However, the high rates of multidrug resistance and ESBL production highlight the necessity for routine surveillance, and early detection strategies to manage such infections effectively.

## Background

Urinary tract infections (UTIs) are a prevalent concern among children, affecting at least 3.6% of boys and 11.3% of girls by the age of 16 [1]. Notably, UTIs can progress to kidney failure, posing serious health risks to children [2]. Among the gram-negative bacteria causing UTIs, *Escherichia coli* (*E. coli)* and *Klebsiella pneumoniae* are predominant pathogens [1]. The rise of antimicrobial resistance (AMR) in *E. coli* and *K. pneumoniae* presents a formidable public health challenge [3]. In 2019, bacterial AMR was attributed for an estimated 1.27 million deaths globally [4]. The consequences of AMR extend beyond clinical outcomes, increasing healthcare costs by prolonging illnesses, extending hospital stays, requiring additional diagnostic tests, necessitating more intensive care, and necessitating the use of more expensive antibiotics. Novel AMR systems driven by natural genetic mutation are emerging and spreading within bacterial population, leading to an increase in multidrug-resistant (MDR) bacteria [5]. The production of beta-lactamases is a common mechanism of antimicrobial resistance, which confers resistance to beta-lactam antibiotics, including cephalosporins and carbapenems [6]. ESBLs were first reported in Germany in 1983 [7] and Nepal in 2006 [8]. Cross-resistance to other non-beta-lactam antibiotics is common in ESBL-producing bacteria, limiting therapeutic choices [9].

While the bacterial AMR profiles vary temporally and geographically, there exists a notable gap in the epidemiological understanding of ESBL-producing *E. coli* and *Klebsiella* spp. among children in Nepal. Surveillance data of this nature holds immense value for guiding evidence-based therapy and improving the management of such infections by healthcare providers. Therefore, this study aimed to elucidate the epidemiology of ESBL-producing *E. coli* and *Klebsiella* spp. in urine samples obtained from children at a tertiary care children's hospital in Nepal.

## Methods

### Study setting, design, and sample population

From August 2016 to February 2017, a prospective cross-sectional study was conducted at the Department of Microbiology, International Friendship Children Hospital in Kathmandu, Nepal. The study included 745 non-repetitive clean-catch midstream urine samples from pediatric patients under the age of 13 with symptoms indicative of UTI. Symptoms included urgency, dysuria, frequency, loss of bladder control, suprapubic tenderness, flank pain, costovertebral angle pain and tenderness, rigors, new or worsening fever, cloudy or foul-smelling urine, and parental reporting of similar symptoms in infants and younger children. Patients receiving antibiotic therapy or had undergone bladder catheterization within 48 h prior to sample collection were excluded. Demographic data including age, sex, and hospital ward were retrieved from hospital records.

### Collection of urine samples

10–15 ml clean catch urine samples were collected using sterile, dry, wide-mouthed, and leakproof containers. For non-toilet trained children and infants, sterile foil bowls were placed beneath the genitalia, or sterile plastic bags were attached. Toilet-trained children provided clean catch voided midstream urine samples. Thorough periurethral cleaning was recommended prior to urine collection to minimize contamination. Noninvasive techniques were exclusively employed for urine collection. Equivocal samples underwent repeat testing.

### Laboratory examinations of urine samples

Macroscopic examination: Urine samples were visually inspected for signs of contamination.

Quantitative culture and identification of isolates: The calibrated loop technique was used for quantitative culture, and isolates were identified as *E. coli* or *Klebsiella* spp. based on Gram's stain, cultural characteristics, and biochemical properties [10].

### Antimicrobial susceptibility testing of *E. coli* and *Klebsiella* spp.

Antibiotic susceptibility testing was performed using the Kirby-Bauer disc diffusion method on Mueller–Hinton agar (MHA) (HiMedia Pvt. Ltd., India). The following antibiotic discs (HiMedia Pvt. Ltd., India) were utilized: amoxicillin (10 μg), cefixime (10 μg), cefotaxime (30 μg), cefpodoxime (30 μg), ceftazidime (30 μg), ceftriaxone (30 μg), ciprofloxacin (5 μg), cotrimoxazole (1.25/23.75 μg), gentamicin (10 μg), imipenem (10 μg), nitrofurantoin (300 μg), norfloxacin (10 μg), ofloxacin (5 μg), piperacillin (100 μg), and vancomycin (30 μg). Interpretation followed the CLSI M100-S26 guidelines, with *Pseudomonas. aeruginosa* ATCC 27853 and *E. coli* ATCC 25922 used as control strains [11]. Isolates resistant to two or more classes of antimicrobial agents were classified as multidrug-resistant (MDR).

### Tests for ESBL production in *E. coli* and *Klebsiella* spp.

ESBL-producing isolates were phenotypically identified following the CLSI M100-S26 guidelines [11].

### Screening of ESBL-producing strains

A zone of inhibition ≤ 25 mm for ceftriaxone, ≤ 22 mm for ceftazidime, ≤ 17 mm for cefpodoxime, and/or ≤ 27 mm for cefotaxime was indicative of confirmation of ESBL [11].

### Confirmation of ESBL-producing strains

ESBL-producing isolates were phenotypically confirmed using the combined disc method. The zones of inhibition were assessed individually for the ceftazidime (30 µg) and cefotaxime (30 µg) discs, as well as in combination with clavulanic acid (10 µg), with the discs positioned 25 mm apart (center to center). Isolates showing a zone size increase of 5 mm or more around one or both of the clavulanate combined discs compared to the antibiotic alone were confirmed as ESBL producers. Control strains, *K. pneumoniae* ATCC 700603 and *E. coli* ATCC 25922 were included for reference [11].

### Data management and statistical analysis

First, Microsoft Excel 2016 (Microsoft Corporation, USA) was used for data management, and then R© for Windows version 4.3.2 (R Core Team, Austria) was used for statistical analyses. Descriptive statistics were presented using percentages. The R package 'gmodels' (version 2.18.1) was utilized to generate the cross-tabulations, and the built-in R function ‘fisher.test’ was used to perform Fisher’s exact test (FET) for the test of independence. Cross-tabulations larger than 2×2 were tested using the Monte Carlo method of FET with 5,000 replicates. Odds ratios (ORs) and confidence intervals (CIs) were used to gauge the strength of the associations. Significant cross-tabulations larger than 2×2 were reparameterized into 2×2 cross-tabulations for the post hoc test. In a post hoc test, FET was utilized to identify specific significant categories. A *p* value of less than or equal to .05 indicated statistical significance. For inferential statistics, the traditional null hypothesis significance testing method based on the *p* value was employed.

## Results

**Fig. 1:**
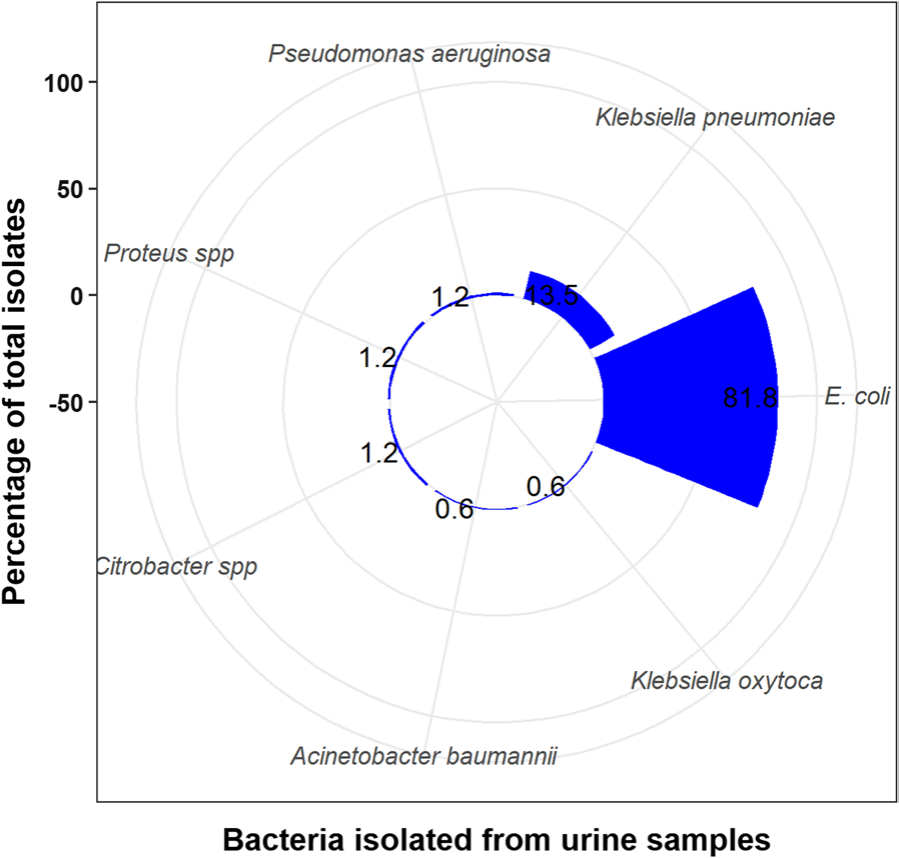
Percentages of bacterial isolates in the samples (*n* = 170)

Out of the 745 samples, bacterial growth was observed in 170 (22.8%) cases. The incidence of growth was similar in samples collected from males and females. A higher proportion of samples from inpatient care settings exhibited bacterial growth, accounting for 36 (30.0%) cases. Despite the absence of a significant association between sex and bacterial growth (*p* value= 1, FET), a significant association was observed between patient care setting and bacterial growth (*p* value= .044, FET). Specifically, the odds of bacterial growth were lower among inpatients compared to outpatients (OR= 0.64). It is important to note, however, that the effect size was relatively small, and the exact strength of the association remains uncertain (95% CI 0.41-1.02) (Table 1). Notably, *E. coli* was the predominant bacterial isolate, accounting for 139 (81.8%) of the identified cases (Fig. 1).

**Table 1:** Distribution of samples

	Growth	OR and 95% CI	*p* value
Sex			
Males (*n* = 245)	56 (22.9%)	1.0 (0.68–1.46)	1
Females (*n* = 500)	114 (22.8%)		
Patient care setting			
Outpatients (*n* = 625)	134 (21.4%)	0.64 (0.41–1.02)	**.044**
Inpatients (*n* = 120)	36 (30.0%)		

OR = odds ratio; 95% CI = 95% confidence interval; boldfaced *p* value denotes significance; percentages were calculated on row totals

**Fig. 2:**
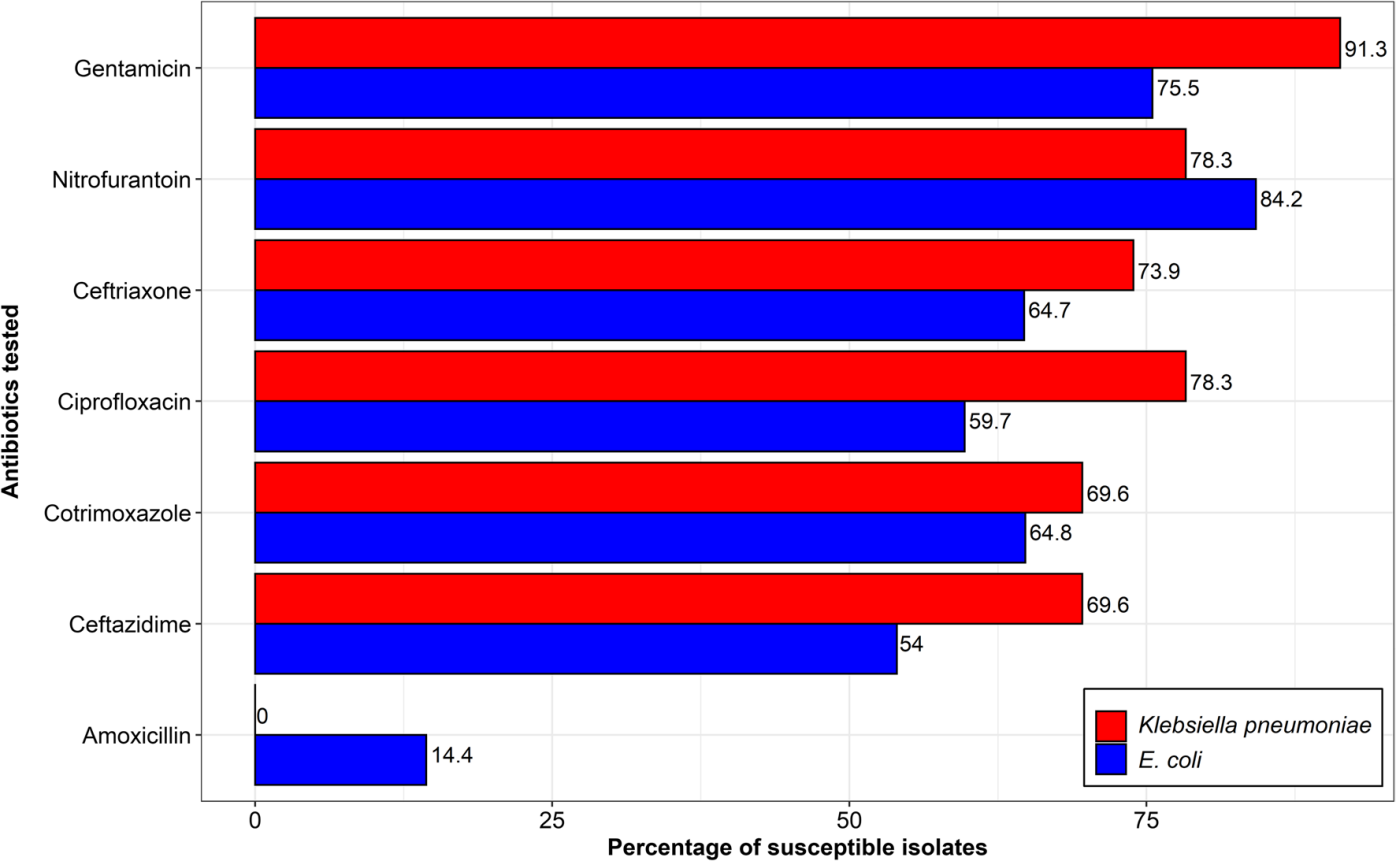
Percentage susceptibility of *E. coli* (*n* = 139) and *K. pneumoniae* (*n* = 23) isolates

*E. coli* showed high susceptibility to nitrofurantoin (117, 84.2%) and gentamicin (105, 75.5%), however, lower susceptibility to amoxicillin (20, 14.4%). Conversely, *K. pneumoniae* showed high susceptibility to gentamicin (21, 91.3%), nitrofurantoin, and ciprofloxacin (both 18, 78.3%), while showing no susceptibility to amoxicillin (Fig. 2).

Of the 163 isolates of *E. coli* and *Klebsiella* spp., 62 (38.0%) were found to be MDR. Among these isolates, 71 (43.6%) tested positive during ESBL screening, yet only 42 (25.8%) of them were confirmed as ESBL producers using the combined disk method. Notably, ESBL-positive cases were predominantly observed in *E. coli*, while *K. pneumoniae* and *K. oxytoca* showed fewer instances. Investigation of the association between ESBL detection and the three different bacterial species revealed a statistically significant association (*p* value = .011, FET). Individual post hoc tests were conducted to assess specific pairs of categories. Notably, the post hoc test further revealed significant associations between the ESBL detection in both *E. coli* and *K. pneumoniae* (*p* value = .010, FET and *p* value = .009, FET, respectively), with estimated odds ratios of 9.54 and 9.04, respectively. However, effect sizes were relatively small, and the exact strength of the associations was uncertain (95% CI  1.45–405.18 and 95% CI  1.37–384.41). No significant associations were observed for *K. oxytoca* (*p* value = 1, FET). This can be attributed to the absence of ESBL producers in *K. oxytoca*, which complicated the statistical results due to extreme values (Table 2).

**Table 2:** Detection of ESBLs using the combination disk method

Organisms	MDR	Screening positive	ESBL producers	*p* value
*E. coli (n* = *139)*	55 (39.6%)	64 (46.0%)	41 (29.5%)	
*K. pneumoniae (n* = *23)*	6 (26.1%)	7 (30.4%)	1 (16.7%)	.011*
*K. oxytoca (n* = *1)*	1 (100.0%)	0 (0.0%)	0 (0.0%)	1**
Total (n = 163)	62 (38.0%)	71 (43.6%)	42 (25.8%)	

The boldface *p* value denotes statistical significance; *p value calculated on first column and fourth column; **p value calculated on second column and fourth column; percentages were calculated on row totals; MDR= multidrug resistance; ESBL=extended-spectrum beta-lactamase; 95% CI= 95% confidence interval

Investigation of the association between ESBL detection and MDR bacteria did not yield statistical significance (*p* value = 1, FET). An individual post hoc test was conducted to assess the sensitivity of the obtained *p* value. We specifically checked the association of ESBL detection in MDR *E. coli* and MDR *Klebsiella* spp. Despite a lack of significance at the conventional level (0.05), a potential association between MDR and ESBLs was suggested by the sharp decrease in the *p* value (*p* value = .14, FET), with an estimated odds ratio of 0.19 and a 95% CI of 0.004–1.61, indicating uncertainty about the true odds ratio (Table 2).

ESBL producers were more prevalent among females (36, 32.7%) and inpatients (12, 35.3%). Significant differences in ESBL prevalence were observed between the sexes (*p* value = .003, FET) (Table 3).

**Table 3:** Distribution of ESBL-producing isolates

	ESBL producer	*p* value	Odds ratio	95% CI
Sex				
Males (n = 53)	6 (11.3%)	**.003**	0.26	0.08–0.70
Females (n = 110)	36 (32.7%)			
Patient care setting				
Inpatients (n = 34)	12 (35.3%)	.186	1.79	0.72–4.32
Outpatients (n = 129)	30 (23.3%)			
Total (n = 163)	42 (25.8%)			

The boldfaced *p* value denotes statistical significance; percentages were calculated for row totals; ESBL = extended-spectrum beta-lactamase; 95% CI = 95% confidence interval

**Fig. 3:**
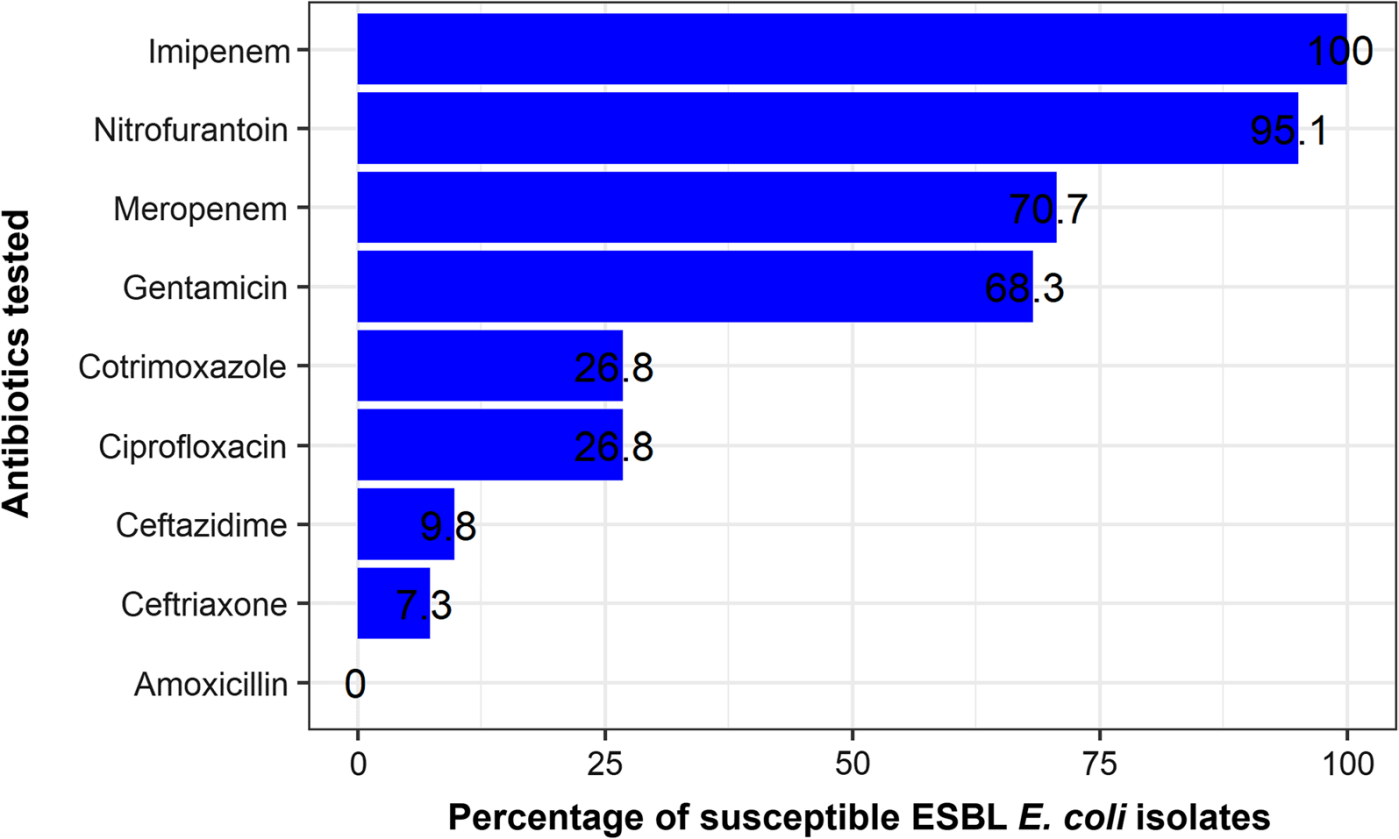
Percentage susceptibility of ESBL-producing *E. coli* (*n* = 41)

Among the 41 ESBL-producing *E. coli* isolates, all exhibited susceptibility to imipenem (41, 100.0%) and nitrofurantoin (39, 95.1%), with none exhibiting susceptibility to amoxicillin (Fig. 3). The single ESBL-producing *K. pneumoniae* isolate exhibited susceptibility to nitrofurantoin, gentamicin, imipenem, and meropenem.

## Discussion

The management of UTIs in children is becoming increasingly complicated due to the rising prevalence of AMR among common pathogens [12]. In this study, only 18.13% of the samples yielded growth in culture, a proportion consistent with findings from other studies conducted in Nepal [13–16]. However, other studies in Nepal reported contrasting results indicating meager proportions [17, 18]. This entails a reevaluation of the existing protocols for pediatric clinical diagnosis of UTIs, focusing microbiological examinations solely on urine samples suggestive of clinical UTIs. Such an approach promises heightened sensitivity and efficacy in clinical diagnosis while conserving considerable resources and curtailing cost. The association of both *E. coli* and *K. pneumoniae* with nosocomial infections in children is a cause for, concern. These organisms normally inhabit the periurethral region as commensals. The development of UTIs occurs when these organisms gain access to and ascend within the urethra [19].

Infections caused by *E. coli* and *Klebsiella* spp. are very common in children. The presence of ESBL in these bacteria is concerning, since it elevates the risk of ESBL-producing organisms among children. In this study, a high proportion (38.0%) of the isolates were identified as MDR. Previous studies conducted in Nepal have reported even higher percentages [13, 16]. Similar results have been reported in the literature from various regions including Nepal [18], India [20], Iran [21, 22], Cambodia [23], and Turkey [24]. However, a systematic review reported lower percentages [25] potentially attributed to variations in the phenotypic detection methods of ESBL production and differences in the study population. The rise in ESBL producers in recent years may be attributed to the increased dependence on third-generation cephalosporins for treating gram-negative infections and questionable antibiotic policies. It is noteworthy that AmpC enzymes, produced by organisms such as *Citrobacter*, *Enterobacter*, and *Serratia*, are not inhibited by clavulanate, potentially leading to false-negative results in ESBL testing. Consequently, the prevalence of ESBL enzymes might be underestimated. Adding an AmpC inhibitor, such as cloxacillin, could enhance the detection of ESBL enzymes in these organisms [26].

All ESBL producers exhibited susceptibility to imipenem. A lower percentage was reported in a study in Nepal [13]. Likewise, other studies in Nepal also reported high susceptibility to imipenem [16, 18]. However, it’s important to note that imipenem, being an intravenous antibiotic, is not recommended as a first-line therapy for UTIs. Although meropenem exhibits moderate susceptibility, it falls under the same class of antibiotics as imipenem, known as carbapenems. The transfer of carbapenemase genes and ESBL genes via plasmids facilitates the rapid dissemination of carbapenemase and ESBL-producing strains. The co-production of carbapenemase and ESBL represents a significant mechanism in the evolving landscape of AMR patterns. Recent years have highlighted a substantial percentage of imipenem-resistant strains, along with co-producing ESBLs, underscoring the urgency of addressing AMR [27] Both ESBL producers and non-ESBL producers exhibited high susceptibility to nitrofurantoin, consistent with findings from studies in Nepal [16–18]. However, nitrofurantoin’s efficacy is compromised by enzymatic degradation, posing challenges in maintaining therapeutic concentrations within body tissues. Consequently, its use is recommended primarily for treating uncomplicated lower UTIs. Moreover, nitrofurantoin’s efficacy is limited against uropathogens other than *E. coli*, since *Pseudomonas* spp., *Proteus* spp., and *Serratia* spp. exhibit intrinsic resistance to this antibiotic [28]. Likewise, *E. coli* and *Klebsiella* spp. exhibited high susceptibility to gentamicin, with the antibiotic exhibiting moderate effectiveness against ESBL producers, consistent with findings from studies in Nepal [16, 18]. In addition, both *E. coli* and *Klebsiella* spp. exhibited moderate susceptibility to co-trimoxazole, an oral antibiotic commonly used to treat pediatric UTIs. Moreover, fluoroquinolones exhibited moderate susceptibility against *E. coli* and *Klebsiella* spp., hence they are not recommended as second-line treatments. Consistent results were reported in a study conducted in Nepal [13]. In addition, both *E. coli* and *Klebsiella* spp. exhibited moderate susceptibility to ceftriaxone and ceftazidime. However, a lower susceptibility to ceftazidime was reported in a study in Nepal [18]. Furthermore, it is recommended to consider ESBL-producing isolates as resistant to these antibiotics, regardless of the susceptibility testing results [11]. This underscores the unreliability of lower-generation cephalosporins as empiric antibiotics. In clinical practice, most children are empirically treated with antibiotics even before culture and susceptibility data are available to prevent complications [29]. The empirical use of gentamicin until the patient’s sensitivity pattern is available may be a prudent approach. Alternatively, nitrofurans remain viable first-line treatments and are cost-effective. In addition, both of these antibiotics are considered safe for pediatric use.

Among the isolates of *E. coli* and *Klebsiella* spp., 38.0% were identified as MDR, while 25.8% were found to be ESBL producers. Given the predominance of ESBL-positive *E. coli* strains, we tested the association between ESBL detection and three different bacterial species. Our statistical analysis revealed a significant association (*p* value = .011, FET). Further exploration through individual post hoc tests uncovered significant associations between ESBL detection in both *E. coli* and *K. pneumoniae* (*p* value = .010, FET and *p* value = .009, FET, respectively), with estimated odds ratios of 9.54 and 9.04, respectively. These findings indicate substantial differences in the odds of ESBL detection in both *E. coli* and *K. pneumoniae*. However, the effect sizes were relatively small, and the exact strength of the associations remained unclear (95% CI  1.45–405.18 and 95% CI  1.37–384.41). We explored the association between ESBL detection and MDR across different bacterial species. Our statistical analysis did not reveal a significant association (*p* value = 1, FET). To delve deeper into this relationship, we conducted an individual post hoc test focusing specifically on MDR *E. coli* and MDR *Klebsiella* spp. However, the results did not reach statistical significance at the conventional level (0.05). Nonetheless, there was a notable decrease in the *p* value, suggesting a potential association between MDR and ESBL detection (*p* value = .14, FET). The odds ratio was estimated to be 0.19 with a 95% CI of 0.004–1.61, indicating uncertainty regarding the true odds ratio. It’s important to note that while Fisher’s exact test provides insights into the overall association between categories based on aggregate counts, it does not address uncertainties or misclassification at the individual level.

Antibiotic resistance emerges from a complex interplay of factors including ineffective antibiotic policies, poor surveillance and compliance measures, unrestricted access to antibiotics, rampant self-medication with poor adherence, suboptimal dosing practices, diagnostic errors, proliferation of low-quality counterfeit drugs, unregulated antibiotic use in agriculture, and indiscriminate antibiotic use without proper diagnosis or identification of the causative agent [30–32]. Antibiotic resistance patterns vary by region and time. The continual evolution of resistant strains to new antibiotics poses significant challenges in identifying effective antimicrobial drugs for treating infections caused by these pathogens. The demand for newer, more expensive drugs, coupled with pronged hospital stays, contributes to escalating healthcare costs. Since this study was confined to a single tertiary setting, further surveillance is imperative to disseminate local resistance profiles. Establishing robust surveillance systems is crucial for guiding antibiotic prescribing, mitigating resistance risks, and optimizing patient care. While empirical therapy plays a pivotal role in avoiding complications, its indiscriminate application without surveillance data can exacerbate antibiotic resistance. Considering local resistance profiles is imperative when prescribing antibiotics empirically. Such an approach will ensure the implementation of the most effective empiric (and definitive) antibiotic therapy for UTIs in children.

Phenotypic detection of ESBLs is a time-consuming and labor-intensive process, often yielding variable degrees of accuracy. Nonetheless, in routine investigations, it serves as a cost-effective alternative to molecular techniques. Understanding the prevalence of ESBLs and other antibiotic resistances in local areas can enhance physicians’ ability to make informed clinical decisions. Future studies should prioritize identifying the risk factors associated with infections caused by ESBLs and other antibiotic resistances.

## Conclusions

The study highlights the commonality of ESBL production among *E. coli* and *K. pneumoniae* isolates from children in Nepal, underscoring the ongoing public health concern. The findings underscored significant resistance to commonly prescribed antibiotics among pediatric patients. Consequently, it is imperative to conduct antimicrobial susceptibility testing before initiating antibiotic therapy in children. If empirical treatment becomes necessary, nitrofurantoin and gentamicin stand out as effective and appropriate empirical choices for treating *E. coli* and *Klebsiella* spp. infections in children. Moreover, these findings underscore the urgent necessity for comprehensive antimicrobial stewardship programs to combat antibiotic resistance.

## Limitations of the study

First, the study was limited to a single site over six-months with a relatively sample size. As a result, the conclusions drawn from the data may not be accurate. Conducting multicenter surveillance involving a larger sample population would bolster the reliability of the findings. Second, we were unable to comprehensively assess the associated risk factors and outcomes of UTIs in children. Third, future research should consider incorporating cohort studies to explore outcomes of antimicrobial therapy and associated risk factors. Fourth, performing antimicrobial susceptibility testing using dilution methods and assessing the minimum inhibitory concentration (MIC) would offer more precise data for monitoring drug resistance. Finally, phenotypic characterization is ineffective in determining the underlying cause of AMR in the population. We were unable to determine the genotypes of the ESBLs among the isolates due to a lack of resources. Genotyping ESBL genes would provide better insight into ESBL production among isolates in the population.

## Data Availability

The complete data set generated and analyzed during the study is already covered in the text. The raw data can be made available upon reasonable request to the corresponding author.

## Abbreviations

AMR: Antimicrobial resistance
ATCC: American Type Culture Collection
CFU: Colony-forming units
CLSI: Clinical and Laboratory Standards Institute
ESBL: Extended-spectrum beta-lactamase
FET: Fisher’s exact test
MDR: Multidrug resistance
MHA: Mueller Hinton Agar
UTI: Urinary tract infection
